# Identification of Medicinally Active Ingredient in Ultradiluted *Digitalis purpurea*: Fluorescence Spectroscopic and Cyclic-Voltammetric Study

**DOI:** 10.1155/2012/109058

**Published:** 2012-03-26

**Authors:** Anup Sharma, Bulbul Purkait

**Affiliations:** ^1^Indian Institute of Technology, Kharagpur, Kharagpur 721302, India; ^2^Department of Biochemistry, Midnapur Medical College and Hospital, Midnapore 721101, India

## Abstract

Serially diluted and agitated (SAD) drugs available commercially are in use with great faith because of the astonishing results they produce. The scientific viewpoint attached to the centuries-old therapy with SAD drugs, as in homeopathy, remained doubtful for want of appropriate research and insufficient evidence base. The conflicting points related to SAD drug mostly related to the level of concentrations/dilutions, use of drug in contradictory clinical conditions compared to the modern system of medicine, identification of medicinally active ingredient in concentrations and dilutions used in commercially available SAD drugs, and lack of laboratory-based pharmacological data vis-à-vis modern medicine. Modus operandi of SAD drug is also unknown. To address some of these issues an analytical study was carried out wherein commercially available SAD drug *Digitalis purpurea*, commonly used in different systems of medicine, was put to test. Various concentrations of commercially available *Digitalis purpurea* were analyzed using analytical methods: cyclic voltammetry, emission spectroscopy, and UV-VIS spectroscopy. These analytical methods apparently identified the medicinal ingredients and effect of serial dilution in commercial preparation of the drugs.

## 1. Introduction

The drug *Digitalis*, an extract of* Digitalis purpurea*, Foxglove plant, is in use since long. Medical practitioners, namely, William Withering, 1785 [1] and Hahnemann, 1803 [2], had introduced the drug in their respective systems of medicine. Out of about thirty known organic compounds of *Digitalis purpurea *only 4–6 are medicinally active components comprising of Digoxin, *Digitoxigenin*, *Digoxigenin*, and *Saponins*. *Digitalis purpurea *derivatives are used in treatment of diseases like heart failure, arrhythmia, neurological diseases and also being tried as antitumor [3]. *Digoxin* (C_41_H_64_O_14_) is 300 times more potent than the powder prepared from *Digitalis purpurea*. It has a molecular weight of 780.95, and the absolute bioavailability of Digoxin intravenous injection is 100%. Time of onset of the effect of Digoxin after intra-venous injection is 5–30 minutes, Patients maintained on Digoxin may also use ultradiluted preparations of the same, ultimately increasing the body burden of Digoxin. Saturated extract of an aqueous ethanol mixture is used to prepare serial dilution of the drug *Digitalis purpurea* and is represented by the suffix “*θ*”. Serial dilutions also are referred to as potencies “1c” or “1” in centesimal scale, which means that one part of the “*θ*” saturated extract of the drug is mixed to make 100 parts of aqueous ethanol mixture and succused. When the same process is repeated 6 times, by adding 1 part of the previous potency in 99 parts of aqueous ethanol mixture and succused, after each stage starting from the first dilution for the next 5 times, “6c” or “6” potency is obtained. Presently, it is not possible to detect the serially ultradiluted drug *Digitalis purpurea* in biological samples. However, attempts are on to find the “evidence base” of serially ultradiluted drugs. Methods to identify the medicinally active ingredient in ultradiluted drugs have been attempted sparingly [4]. The investigation of homeopathic drugs is extremely interesting and challenging and from that point of view shows novelty. *Digitalis purpurea* is readily available commercially, in serial dilutions prepared with aqueous alcohol (91.4%) as diluent. The serial dilution 12c of the drug, at a ratio 10^−24^, has a 60% probability of containing one molecule of original material if one mole of the original substance was used. Digitalis-like toxicity (*Digitalis intoxication*) results from an overdose of the drug, consumption of *Digitalis purpurea* plant products, and from toad skin (another animal source: butterflies) [5]. *Digitalis *intoxication causes anorexia, nausea, vomiting, and diarrhea, jaundiced or yellow vision, appearance of blurred outlines, reduced heart rate, and weight loss. It is also necessary to evaluate the quality of the drug in solutions/dilutions. This motivated us and necessitated detection of *Digitalis purpurea* in serial dilutions. Attempts had been made to detect Digoxin in solutions by various methods [6].

The primary aim of this work is to detect the presence of *Digitalis purpurea *(DP), Digoxin, and/or Digoxin*-like* substance in serial ultradilutions using fluorescence spectroscopy and cyclic voltametry for comparison of the components of *Digitalis purpurea*, which has intrinsic fluorescence.

This original research work offers additional data for improving standardization of ultradiluted drugs in complementary/alternative system of medicine. Two analytical methods, fluorescence spectroscopy and cyclic voltametry (CV), of the ultradiluted drugs *Digitalis purpurea* vis-à-vis Digoxin (0.25 mg/mL) were carried out for detection of the Digoxin-like substance.

**Figure 1:**
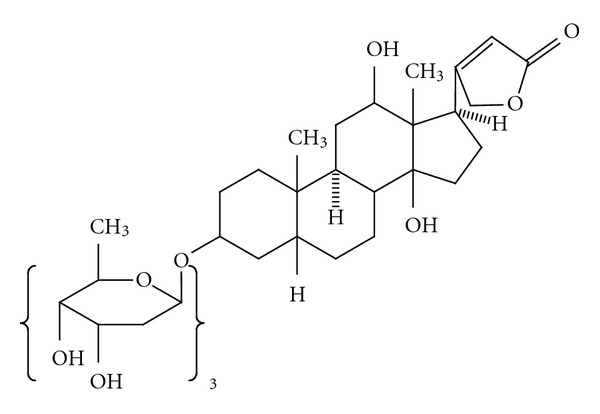
Structure of Digoxin.

The methods reported here are highly selective, sensitive, stable, and reproducible. The structure of medicinally active ingredient *Digoxin* is given in Figure 1. The compound is known to be hydrophobic in nature, but the sugar moiety attached to the compound changes its solubility. It is found to be somewhat soluble in chloroform in its purest form.

## 2. Methodology

The drug *Digitalis purpurea* used in this work in potencies/ultradilutions *θ*, 6, 30, and 200 were procured from M/s Hahnemann Publishing Company Pvt Ltd, Kolkata, India. It was prepared with contaminant free, for example, acetone-free aqueous alcohol (91.4% ethanol and glass double distilled water mixture). The drugs were manufactured as per Indian Homeopathic Pharmacopoeia. The commercially available Digoxin with concentration 0.25 mg/mL (Samarth Pharma) was procured from the local medicine vendor for the present studies. Rectified spirit/ethanol manufactured by Bengal Chemicals & Pharmaceuticals, India, was used.

All the experiments were carried out to ensure reproducibility. For constrains attempt to detect the upper limit could not be carried out, although we carried out the voltammetric measurements using saturated extracts of the drug. It is well known that the Lambert-Beer Law is valid for dilute solutions and the fluorescence intensity is linearly related to concentration of dilute solutions.

The UV-VIS and emission spectra were measured using a Shimadzu (model no. UV-1601) spectrophotometer, and a Spex-Fluorolog-3 (model no. FL3-11) spectrofluorimeter respectively. The UV-VIS maximum for Digoxin is calculated to be at 255 nm and, in another experimental study, published earlier, an absorption maximum was found to be 251 nm by the authors [7]. Emission spectra were obtained by analytical fluorescence spectroscopy of natural product *Digitalis purpurea *(in ultradilutions) and its purest derivative component Digoxin.

All the voltammetric measurements were carried out using a computer-controlled CHI643B electrochemical analyzer attached with a Faraday cage/picoampere booster (CH Instruments, Austin, TX). A two-compartment three-electrode cell with glassy carbon (GC) and a polycrystalline gold (Au) working electrode (2 mm dia, BAS, USA), a platinum wire auxiliary electrode, and an Ag/AgCl (3 M KCl) reference electrode were used in the measurements.

## 3. Electrode Modification for Voltammetric Measurements

Polycrystalline Au was polished well with alumina powder (0.06 *μ*m) and sonicated repeatedly in water for 8–10 min. The polished Au electrode, of geometrical surface area 0.031 cm^2^, was cleaned electrochemically by cycling the potential between—0.2 and 1.5 V in 0.25 M H_2_SO_4_ at the scan rate of 10 V/s for ~10 min, until the characteristic cyclic voltammograms for a clean Au electrode were obtained, and the same was used as working electrode. All the experiments were carried out in 0.1 M Na_2_HPO_4_ (pH 11). *Digitalis purpurea* in serial dilution and its purest derivative *Digoxin* were used in all the following studies. The voltammograms of *Digitalis purpurea* Q, 6C, 30C, and 200C dilutions, and Digoxin were obtained using pairs of Au electrodes at pH 11, at scan rate of 100 mV/s. 100 *μ*L of samples was used.

## 4. Result and Discussion

**Figure 2:**
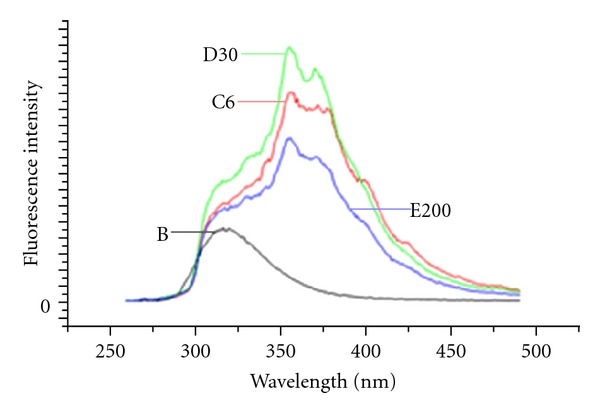
Emission spectra obtained by analytical fluorescence spectroscopy of natural product *Digitalis purpurea* derivative having the purest derivative component *Digoxin *(B) showing emission maxima at 318 nm, showing a relative intensity of 4.5 × 10^6^. C6: *Digitalis purpurea *6 having emission maxima at 357 nm and 374 nm showing a relative intensity of 13 × 10^6^ and 12 × 10^6^. D30: *Digitalis purpurea* 30 having emission maxima at 357 nm and 374 nm showing a relative intensity of 16 × 10^6^ and 14.5 × 10^6^. E200 = *Digitalis purpurea *200 having emission maxima at 357 nm and 374 nm, showing a relative intensity of ~10 × 10^6^ and 9 × 10^6^.

The fluorescence is likely to be due to *α*, *β*-unsaturated lactone present in the compound. The fluorescence spectrum of the compound is taken at different dilutions in aqueous ethanol. In all the emission studies, the samples were excited at 255 nm. The emission spectra of different dilutions are shown as C, D, and E, and purest medicinally active ingredient Digoxin is shown as B in Figure 2. These fluorescence spectra revealed that *Digoxin* component shows a structureless fluorescent with emission maxima at 318 nm with a relative intensity of 4.5 × 10^6^. The emission spectra of *Digitalis purpurea *6 are shown in Figure 2 as C6. In this case the structureless emission is found to be modified with a semistructured emission with maxima at 357 nm and 374 nm and further a shoulder at about 318 nm remained, which could be due to the original B (*Digoxin*). The appearance of additional bands at 357 and 374 nm may be ascribed to loss of self-quenching or dissociation of component substance due to serial dilution, and these bands showed an increase in intensity with subsequent dilutions up to a certain level [8].

The serial sequential dilutions of the natural product at 6c, 30c, and 200c show emission maxima at 318 nm rather than the structured emission as observed with purest digitalis. This could arise due to the interference caused by other inclusions in the samples.

Although, with the dilution, the peak intensity of the compounds should gradually increase or decrease, the gradual increase in emission with dilution is due to reduction of self-quenching of fluorophores as a result of concomitant dilution. The decrease of emission with still further dilution is caused by simple dilution effect. Since the medicinally active ingredients in serial dilution of drug are not detectable by the common analytical methods in vogue, the results cannot be compared with usual standard methodologies.

**Figure 3:**
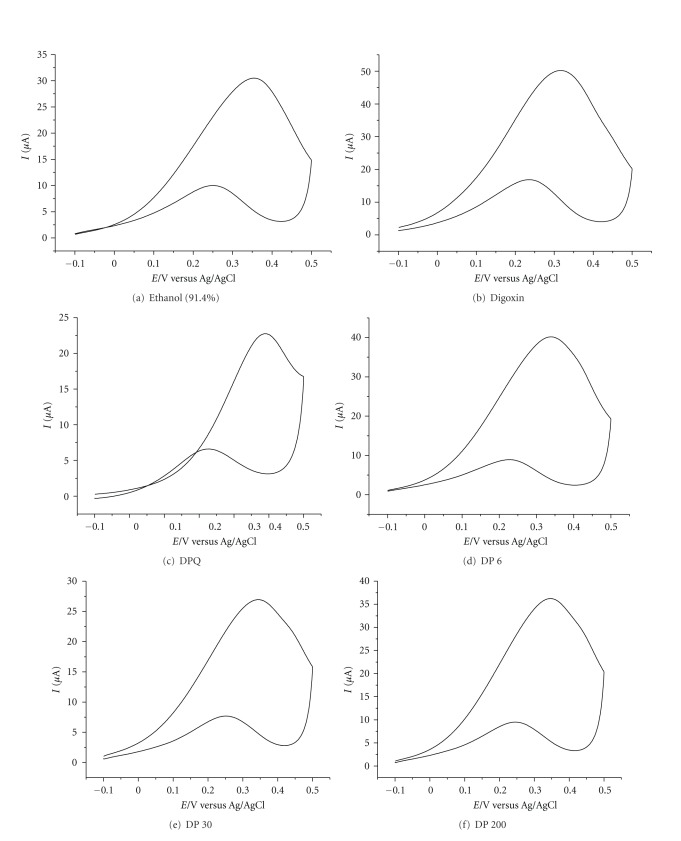
(a) Voltammograms of ethanol (91.4%), (b) Digoxin, (c) *Digitalis purpurea θ*, (d) *Digitalis purpurea* 6, (e) Digitalis purpurea 30, and (f) Digitalis purpurea 200.

The Au voltammograms of the drug samples are shown in Figures 3(a)–3(f).These voltammograms clearly indicate the presence of a redox pair showing distinct peak potentials, at *∼*0.25 and 0.35 volt with Au electrode and *∼*0.35 and 0.45 volt with bare Au electrode, respectively. The same redox pair is seen with the medicinally active pure derivative *Digoxin*. Perfect reversible cyclic voltammograms are obtained with the Au electrode. Interestingly, for both Au and bare Au cases we got the same potential separation for the cathodic and anodic peak. The cyclic voltammograms indicate the presence of the peak for *Digoxin *(purest *medicinal* derivative of *Digitalis purpurea*) at the same level as that of serially ultradiluted drugs. This indicates the presence of *Digoxin-like* substance in the ultradiluted samples of the drug. As the structure in Figure 1 indicates, the five-member moiety containing *α*, *β*-unsaturated carbonyl moiety is responsible for accepting one electron during reduction in the cyclic voltammogram.

## 5. Conclusion

Fluorescence spectroscopy and cyclic voltametry both can be used to identify *Digitalis purpurea *and its derivatives, in commercially available serially ultradiluted solutions.

## 6. Limitations of the Study

The study has several limitations. Serial ultradilutions beyond 200 have not been tried. Dilutions made out of *Digitalis purpurea θ* were not tried as the same are not available commercially. Medicinally active ingredients of serial ultradilutions of drugs are difficult to detect analytically in laboratory. Commercially available medicines were only tried. Quantitative estimation of the active ingredients of the drugs has not been made. Drugs in serial ultradilutions are not detectable in biological specimen, bringing limits to the design of the research. The glassy carbon electrodes were used initially for cyclic voltammetry, which did not show identification/detection of the drug in serial ultradilution. The obtained data/results with glassy carbon and Au electrodes are not presented in this paper.

## 7. Future Scope

A comparative analysis of levels of proportional bulk concentration using the Randle-Sevcik relationship may be performed. These techniques may prove valuable in standardization of diluted drugs as used in complementary and alternative medicine. Fluorescence spectra and the redox patterns indicate changes arising out of the serial dilution, which may form the basis of future studies. Both these techniques may be useful for the point-of-care services in cases where patients are being “Digitalized,” in cases of “poisoning with Digitalis,” and for toxicological studies of* Digitalis purpurea* in nonbiological samples and its active derivatives. This would enable physicians to take posological and antitoxic measures.
